# 

**Published:** 1874-04-15

**Authors:** 


*From W. B. Keen, Cooke & Co., Chicago.*

A Manual of Toxicology. By John J. Reese M.D. Philadelphia: J. B. Lippincott & Co., 1874.

A Treatise on Therapeutics, comprising Materia Medica and Toxicology. By H. C. Wood, Jr., M.D. Philadelphia: J. B. Lippincott & Co., 1874.

